# Chicago Society of Ophthalmology and Otology; Meeting June 8

**Published:** 1886-12

**Authors:** 


THE CHICAGO SOCIETY OF OPHTHALMOLOGY AND OTOLOGY *met on June 8th* at the Tremont House. E. L. HOLMES, M.D., in the chair.

Dr. H. M. Starkey presented three cases of congenital ectopia lentis, with the following histories:

1. Mrs. Joseph Peltier, widow, age forty-four. Congenital ectopia lentis of both eyes, and the mobility of the eye-balls slightly diminished. Tension normal in both eyes. Right eye—the lens was cataractous. Vision=perception of light. Left eye—Vision=20-200 with + 5.5 D.=20-50. Ophthalmoscopic examination showed the fundus to be normal. Accommodation was nil. Lenses dislocated upwards and backwards. The irides and the lenses were quite tremulous. The patient was given + 5.5 D. for the distance, + 9 D. for close work.

2. Gertrude Peltier, a daughter, age sixteen. Double ectopia lentis, backwards and upwards, with tremulous irides and lenses. Her general health was good. There was slight photophobia. Vision—right eye, 4-50 with + 7 D.=

20-30; left eye, 2-50 with + 7 D.=20-70, and the accommodation of both eyes=0. Tension was normal. On ophthalmoscopic examination the fundus was found to be normal. She was given + 7 D. oc. u. for distance, + 12 D. oc. u. for reading.

3. Aaron Peltier, a son, age seven. Both lenses were dislocated upwards and backwards. Tension was normal. Ophthalmoscopic examination showed the same. Mobility was impaired, irregular and discordant. Accommodation=0. Vision—right eye=20-120 with + 8 D.=20-60; left eye=20-120 with + 8 D.=20-50. Prescribed + 7 D. oc. u. for distance. Another son, aged sixteen, had been examined by Dr. Boerne Bettman, who found both lenses likewise dislocated (upwards). The boy's vision was materially improved by a + 8 D. lens for each eye.

After an examination of these interesting cases by the members of the society, Professor W. T. Montgomery read the following papers:

OPERATION BY LIGATURE FOR ENTROPION OF LOWER LID.

About two months ago, an old man came under my care who was suffering from complete inversion of all his eyelids and marked narrowing of the palpebral fissures. These conditions had existed for a considerable time, so that the patient was almost blind from pannus. Under ether I made canthotomy on both eyes and Hotz's operation on both upper lids, intending to make this latter operation on both lower lids. Owing to the tediousness of the Hotz operation and to the fact that it is not nearly so effectual in correcting entropion of the lower as of the upper lid, it occurred to me to try the effect of a ligature introduced as follows: A strong curved needle was threaded with number 9 surgeon's silk and introduced at a point four to five millimeters below the puncta and passed deeply through the tissues of the lid and brought out at a point four to five millimeters below the outer extremity of the lid. The included tissue was then firmly ligated. The inverted lid was completed everted but the ligation produced such an unsightly puckering of the lid that I was tempted to remove the ligature, but did not. Both lower lids were treated in the same manner. The ligatures were removed on the third or fourth day, or as soon as suppuration began, and the unsightly puckering soon disappeared, the lids assuming a natural position. This patient remained under observation about one month and there was no appearance of return of his entropion. I have made the operation about fifteen times with very satisfactory results so far. It has only failed in correcting the entropion in one case, and this was such an aggravated case that I was not surprised at failure at the first attempt. I ligated again, and the lid is now everted. The effect of the ligature is modified by the amount of tissue included. It is too early yet to speak assuredly of the ultimate success of this operation. I have not the temerity to call it new. I can only say it is new to me, and being so very simple, if it is even as successful as other operations in correcting entropion of the lower lid, it commends itself.

He then exhibited a patient with the following history:

Eugene Darust, age twenty-seven, baker, ten years ago received a blow on the left eye with the fist or some blunt instrument which knocked him down. In falling, the left temporal region struck the flat surface of a stone. Considerable swelling and ecchymosis of the lids followed these injuries, but soon began to clear up, and in a short time there was only a slight thickening of the lids remaining. This slight thickening did not noticeably change until about three years ago, when it began to gradually increase and the surface to assume a knotty appearance. The trouble, though slowly increasing, had not given the patient any inconvenience, except its unsightly appearance, up to the time I first saw him, on December 4th, 1885. At this time the upper lid was so much thickened that there was almost complete ptosis. The external surface presented a markedly nodular and purple appearance, due to varicose enlargement of blood-vessels; on the inner surface of the upper lid near the external angle was found a varicose nodule, the size of a pea. The entire palpebral conjunctiva presented an engorged appearance. The lower lid was also considerably thickened by the varicose engorgement. Decided pulsation was felt over the entire upper lid and extending into the temporal region on this side, but was most marked over the outer angle of the orbit and over the supraorbital foramen. Firm pressure at these points arrested pulsation in the lid and lessened the vascular engorgement. I informed the patient that I thought an operation, ligating the principal branches of the anterior temporal artery and the supraorbital artery, with the free use of the electrolysis needle, would relieve him; but was not certain of effecting a cure by these means, though I endeavored to impress upon the patient the importance of the affliction and urged the immediate necessity of the operation. I did not see him again until May 9th.

He then stated that three weeks previous the noduli on the inside of the lid ruptured and he almost bled to death before the hemorrhage was stilled. Dr. J. J. M. Angear, who was called to see him at the time, has since informed me that he attempted to ligate the main branch of the temporal subcutaneously, but as this did not check the bleeding he ligated the ruptured vessel on the inside of the lid. Dr. Angear was of the opinion that the varix was largely supplied by branches of the lachrymal artery, and that ligation of the common carotid would have to be made. At the time of the second visit of the patient the condition of the lids was not materially changed, but the patient was still anæmic and weak from loss of blood. I advised the operation which had been urged in the first place, but warned him that it might not prove successful, and in that case ligation of the common carotid would have to be made. The patient acquiesced. On May 11, under ether, four subcutaneous ligatures, one including the pulsating branches of the supraorbital, and three along the course of the temporal branches, were applied. The first of these was introduced at the outer rim of the orbit, and the other two at intervals of one-fourth of an inch towards the temporal region. After the sutures were introduced pulsation was only detected at the outer extremity of the lid in a small area. A fine electrolysis needle was then inserted about a dozen times in different directions through the engorged tissues of the upper lid. Cold water dressings were applied, and the patient was ordered to be kept quiet in his room and friends were warned of the possibility of secondary hemorrhage. Considerable reaction followed the operation. The ligatures had all come away by the end of twelve days without any hemorrhage, and by the end of three weeks the swelling had subsided to a degree less than existed before the operation. The surface of the lid is now smooth. Pulsation is still felt over the outer rim of the orbit and the eye-lids are still much thickened. On June 1 electrolysis was again freely used without employing an anæsthetic, and but little reaction followed.

At present, June 8th, the lids have not entirely recovered from the last operation, but I think it is safe to say that it will be followed by further improvement.

It is still a question as to whether a cure will be effected by the means instituted. I present the patient for your inspection and advice. I am encouraged to give further trial to electrolysis. We all know that ligation of the common carotid is a hazardous operation, and is not always successful in curing aneurisms about the orbit. There is no doubt about the aneurism in this case, starting from the injury of ten years ago, and it is my opinion that the injury was primarily directed to the branches of the middle temporal. It was upon this opinion that my hope of benefiting the patient by the treatment detailed was based.

